# Initiation, Establishment, and Maintenance of Heritable *MuDR* Transposon Silencing in Maize Are Mediated by Distinct Factors

**DOI:** 10.1371/journal.pbio.0040339

**Published:** 2006-09-12

**Authors:** Margaret Roth Woodhouse, Michael Freeling, Damon Lisch

**Affiliations:** Department of Plant and Microbial Biology, University of California Berkeley, Berkeley, California, United States of America; Cold Spring Harbor Laboratory, United States of America

## Abstract

Paramutation and transposon silencing are two epigenetic phenomena that have intrigued and puzzled geneticists for decades. Each involves heritable changes in gene activity without changes in DNA sequence. Here we report the cloning of a gene whose activity is required for the maintenance of both silenced transposons and paramutated color genes in maize. We show that this gene, *Mop1 (Mediator of paramutation1)* codes for a putative RNA-dependent RNA polymerase, whose activity is required for the production of small RNAs that correspond to the *MuDR* transposon sequence. We also demonstrate that although *Mop1* is required to maintain *MuDR* methylation and silencing, it is not required for the initiation of heritable silencing. In contrast, we present evidence that a reduction in the transcript level of a maize homolog of the nucleosome assembly protein 1 histone chaperone can reduce the heritability of *MuDR* silencing. Together, these data suggest that the establishment and maintenance of *MuDR* silencing have distinct requirements.

## Abstract 2

Silencing of the transposon system*Mu* in maize is maintained by*Mop1* (necessary for the presence of*MuDR* small RNAs). This silencing is mediated by a histone chaperone NAP1 ortholog.

## Introduction

Epigenetic variation involves heritable changes in gene activity in the absence of changes in DNA sequence. These changes are a characteristic feature of some developmental programs, where epigenetic states of gene activity can be maintained through mitotic cell divisions [1,2]. Variations in epigenetic states can also persist through meiosis, resulting in non-Mendelian patterns of inheritance. This form of epigenetic variation has been well documented over the years, particularly in maize, but it is only recently that we have begun to understand the mechanism that makes it possible. This knowledge is now informing our understanding of two phenomena that have intrigued geneticists for decades: paramutation and transposon silencing.

In paramutation, a paramutagenic allele of a gene can heritably alter the expression of a second paramutable allele of the same gene. In many cases, the altered allele can then itself become paramutagenic [3]. This phenomenon, which does not involve changes in DNA sequence, has been best studied in maize through the use of alleles of various color genes that undergo paramutation, including *r1, b1, pl1,* and *p1* [4]. The molecular mechanism that makes paramutation possible has been enigmatic. However, in each case where paramutagenic activity can be mapped to a specific region, it is associated with repeated sequences whose copy number has a direct effect on the degree of that activity [5–7]. To date, no evidence for RNAs that can trigger paramutation has been found, and it has been an open question as to whether RNA is directing this process.

Like paramutagenic alleles, most transposons contain tandem or inverted repeats and can trigger heritably silencing [8]. Indeed, Barbara McClintock, who discovered transposons in maize in the 1950s, spent several decades exploring the phenomenology of transposon silencing and reactivation [9]. It is clear from those and subsequent experiments in a number of plant and animal species that transposons and other repetitive elements are particularly prone to epigenetic silencing [10]. In fact, it has been hypothesized that epigenetic silencing arose as a mechanism to inactivate invasive DNA [11]. Certainly, given the mutagenic potential of transposable elements, it is clear that transposon inactivation has become a primary function of gene silencing in eukaryotes [12].

Repeated elements, whether in paramutable alleles or in transposons, can potentially trigger silencing if transcription of those repeats results in the production of double-stranded RNA (dsRNA), which can then be processed into small interfering RNAs (siRNAs) [13]. Mutations in a number of the genes involved in RNA interference (RNAi) in both plants and animals are associated with transposon activation [14–16]. Not only are siRNAs resulting from the processing of dsRNA associated with post-transcriptional degradation of target mRNAs, but they are also implicated in transcriptional silencing of target genes via DNA methylation and histone modification [17,18].

In plants, it appears that RNA silencing pathways have become functionally diversified, such that there are distinct (albeit overlapping) mechanisms for recognition and processing of various aberrant RNAs [19]. The RNAi pathway that appears to be specifically associated with transposon and heterochromatic repeat silencing involves several components. In *Arabidopsis,* these include (but are certainly not limited to) DICER-LIKE3 (DCL3), ARGONAUTE 4 (AGO4), RNA-dependent RNA polymerase 2 (RDR2), and the components of RNA polymerase IV [19–22]. This pathway appears to be functionally distinct from that which is involved in microRNA processing, which also centers around the production and use of small RNAs. Thus, although mutations in *DCL3* and *RDR2* eliminate small RNAs from some transposons [19], they have no effect on microRNA accumulation; the same is true for *Ago4* [22,23]. The available evidence suggests that these factors cooperate to maintain and/or initiate heterochromatic silencing of many endogenous repeated elements [21,24].

One example of an inverted duplication that can reliably and heritably silence a transposon has been identified. This locus, called *Mu killer (Muk)*, is a variant of the *MuDR* autonomous transposon in maize that has duplicate portions of that element joined in an inverted repeat orientation [25]. *Muk* produces a long dsRNA that triggers the rapid processing of normal *MuDR* transposon transcript into small RNAs. This is followed by methylation and transcriptional inactivation of *MuDR*. The two genes encoded by *MuDR* appear to be silenced via distinct mechanisms: the *Muk* transcript only shares homology with the transposase *mudrA,* and during silencing, it is *mudrA* small RNAs that are amplified, and it is the full-length *mudrA* transcript that is targeted for degradation [25]. By the immature ear stage of F1 plants carrying both *MuDR* and *Muk, mudrA* is transcriptionally silenced [8].

The second gene encoded by *MuDR, mudrB,* is required for transposon insertional activity [26,27]. When *Muk* initiates the heritable silencing of an active *MuDR* element, the silencing begins at *mudrA* and eventually spreads in *cis* to the *mudrB* gene, which is not directly targeted by *Muk* [25]. Silencing of *mudrB* begins with the production of only nonpolyadenylated transcript in plants that carry both *Muk* and *MuDR*. In subsequent generations, all transcript from *mudrB* is lost [8], and its terminal inverted repeat (TIR) becomes methylated [27].

Both *Muk*-induced silencing of *MuDR* and paramutation involve directed and heritable epigenetic changes in gene expression, raising the possibility that these phenomena have common requirements. This idea was confirmed with the discovery of a gene that is required for both paramutation and *Mu* transposon methylation [28]. This mutation, *mop1 (mediator of paramutation1)*, causes paramutated alleles of the *b1* gene to express at a high level, and it prevents the process of paramutation at this and several other paramutable loci in maize [29]. *mop1* mutants also reverse *Muk-*induced silencing of *mudrA* after several generations in a *mop1* mutant background [27], and these mutants can also reactivate silenced transgenes [30]. The *Mop1* wild-type allele is required for the default methylation that occurs at nonautonomous elements in the absence of the transposase and is also required to maintain methylation of the TIR adjacent to the *mudrA* gene in *Muk*-silenced *MuDR* elements. It is not required for methylation of restriction sites at the *mudrB* TIR, and after multiple generations in a *mop1* mutant background, *mudrA,* but not *mudrB,* becomes transcriptionally active [27].

In addition to containing *MuDR*, all maize lines examined contain *hMuDR*, or heterologous *MuDR* elements, which are paralogs of the autonomous *MuDR* element. These elements appear largely inactive; they express only small amounts of largely nuclear localized transcript [31], they do not transpose, and they do not cause transposition of nonautonomous elements [32]. Relative to an active *MuDR* element, these elements exhibit decreased DNaseI hypersensitivity (Lisch D, unpublished data). Because many of these elements share high homology to *MuDR*, including promoter regions, it is likely that these elements were once active and have become epigenetically silenced. Thus, in addition to any active *MuDR* elements a given maize line has, the line also contains multiple previously active elements that have presumably become permanently silenced. Given the vast numbers of transposons resident in most genomes and the relatively modest rate of naturally occurring insertional mutations, the silenced state is probably the normal condition for the majority of elements.

Here we describe the cloning and characterization of *mop1* and its role in *Mutator* silencing. We show that *Mop1* is an ortholog of *RDR2* in *Arabidopsis*, confirming recent work by the Chandler laboratory [33]. We find that in *mop1* mutants, small RNAs homologous to *MuDR* transcripts are lost and *Mu* transposon terminal inverted repeats are hypomethylated, consistent with a requirement for *rdr2* activity in the production of small RNAs and subsequent DNA methylation of transposon or transposon-derived target sequences [19,20]. However, *mop1* mutants do not prevent *Muk* silencing of *MuDR,* consistent with the observation that *Muk* produces a double-stranded transcript and has no requirement for RDR activity. Conversely, we provide evidence that maize homologs of *NAP1 (nucleosome assembly protein 1)* genes are specifically involved in the establishment of heritable *MuDR* silencing, but may not be involved in the maintenance of silencing once it has been established.

By using a genetic approach, we demonstrated that two genes, *Mop1* and *NAP1,* play distinct roles in *Mutator* silencing by *Muk*. The mode of action of each of these mutants, along with what is known about *Muk,* make it possible to propose a tentative model for the initiation, establishment, and maintenance of *Mutator* transposable element silencing in maize.

## Results

### *Mop1* Is an RDR

#### The *mop1–1* allele is the result of a *Mu* transposon insertion.

**Figure 1:**
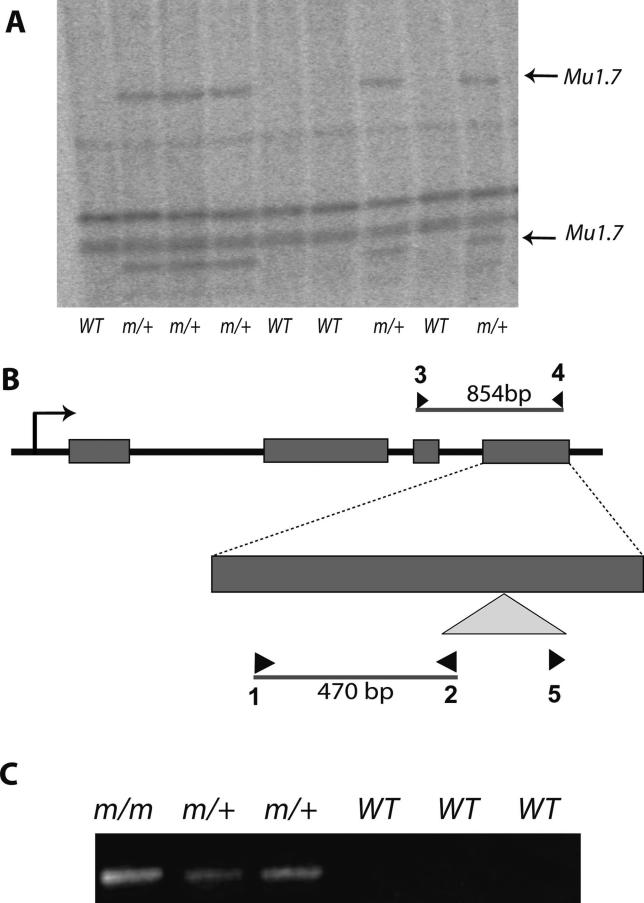
Cloning of the *Mop1* Gene (A) Southern blot depicting a family segregating for either wild-type (WT) or *mop1–1* heterozygous individuals (m/+). Individuals were genotyped using the SSR marker umc1541, which has been previously mapped to within one centimorgan from the *mop1* locus. Arrows denote both *Mu1.7* transposon elements that are only present in individuals containing the *mop1–1* allele. The two other bands in the middle of the blot found in all lanes denote the *Mu1* element in the *a1-mum2* allele that is present in all individuals in this family. DNA was digested with *Nco*I restriction enzyme. (B) Depicted is a map of *ZmRDR2* (not to scale). The *Mu* insertion is depicted here as a gray inverted triangle. Primers (indicated as arrows) correspond to the canonical sequence of ZmRDR2/RDR101 exon 4 (primer 1) and the *Mu1.7* terminal inverted repeat sequence (primer 2). Primers flanking the insertion used for RT-PCR portrayed in figure 2 are designated primer 3 and primer 4. The indicated sizes are those obtained from amplification and sequencing of these products. Primers used to amplify sequences 3′ of the insertion were primer 4 and primer 5. The region sequenced in *mop1–1, mop1–2,* and minimal line wild-type *Mop1* is indicated by the black bar above exon 2. (C) PCR of a family segregating for *mop1–1* homozygote (m/m), heterozygote (m/+), and WT individuals using primers corresponding to *ZmRDR2* exon 4 and *Mu1.7* as depicted in Figure 1B. Individuals carrying the *mop1–1* allele (m/m and m/+) give rise to the expected 470-bp amplicon, indicating the presence of a *Mu1.7* insertion at the mutant allele. Conversely, the WT individuals do not give rise to this amplicon, suggesting that the *mop1–1* allele is the result of a *Mu1.7* insertion in exon 4 of *ZmRDR2*.

While examining families segregating for *mop1–1/+* and homozygous wild-type plants, we observed two *Mu1.7*-homologous fragments that were only present in *mop1/+* individuals (as determined by simple sequence repeat [SSR] mapping; see Materials and Methods) (Figure 1A). Given that the *mop1–1* allele arose in a *Mu*-active line [29], we hypothesized that the *mop1–1* allele may contain a *Mu1.7* insertion. Because *mop1* had been mapped by the Chandler laboratory [33] by using SSRs umc1541 and bnlg1018 from the Maize GDB database (http://www.maizegdb.org/), it was possible to examine the region in rice that is syntenous to markers closely linked to *mop1* in maize. Using the rice public database Gramene (http://www.gramene.org/), we found one candidate gene on rice chromosome 4 (Os04g39160) that is homologous to *RDR2* in *Arabidopsis*. We obtained a partial sequence of the maize putative homolog of this gene (designated here as *ZmRDR2*) by BLASTing the rice DNA sequence against the National Center for Biotechnology Information database (http://www.ncbi.nlm.nih.gov/) and ChromDB (http://www.chromdb.org/) and found a maize express sequence tab (CL3242_1) and a contig sequence (RDR101), which share 99% sequence identity to each other in the coding region. From these sequences, we designed a pair of primers, one from *ZmRDR2* and one from the *Mu1.7* sequence (Figure 1B, primers 1 and 2), to look for an amplification product that was only present in individuals that were either homozygous (as evidenced by demethylation of *Mutator* TIRs and high levels of *B'* expression) or heterozygous (as evidenced by SSR genotyping) for the *mop1–1* allele, which was detected (Figure 1C). Sequencing of the amplification product revealed that the insertion was in exon 4 of *ZmRDR2* (Figure 1 and Figure S1). Restriction digests with several different enzymes resulted in two cosegregating fragments on Southern blots, whereas others resulted in a single, larger fragment that hybridized to our probe with greater intensity (unpublished data). Based on these observations, we hypothesize that the insertion is a compound element that consists of two *Mu1.7-*homologous elements, although we have been unable to amplify the entire element, presumably because of its complex structure. However, we have sequenced both ends of the element along with flanking sequence. As expected, the insertion is flanked by *Mu* terminal inverted repeats and a 9–base pair (bp) target site duplication, consistent with a genuine *Mutator* insertion allele (Figure S1).

**Figure 2:**
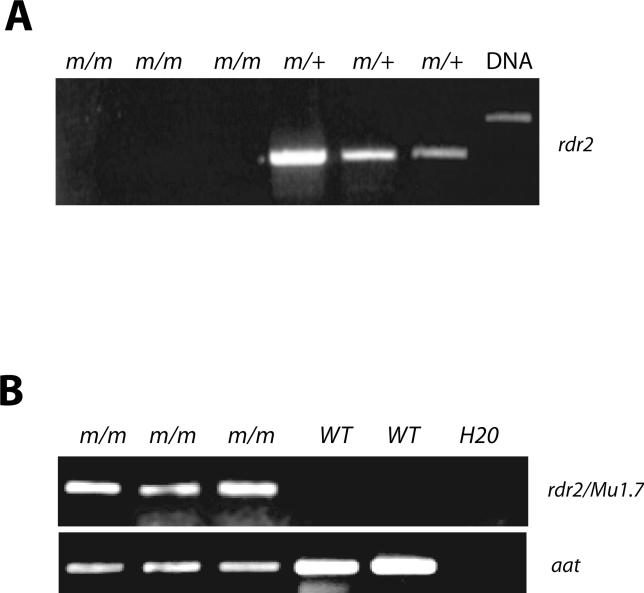
The *ZmRDR2* mRNA Transcript Is Altered in *mop1–1* Mutants (A) RT-PCR of a family segregating for the *mop1–1* allele. Primers used were those corresponding to the *ZmRDR2* sequences and that spanned intron 3 as well as the *Mu1.7* insertion in exon 4 (primers 3 and 4 in Figure 1). Individuals carrying the WT allele (m/+) give rise to an 854-bp cDNA amplicon (as shown in Figure 1B); those that are *mop1–1* homozygous (m/m) do not, suggesting that the *ZmRDR2* RNA transcript is impaired in *mop1–1* homozygous mutant individuals. The DNA control sample gives rise to a 1335-bp amplicon. (B) RT-PCR of WT and *mop1–1* homozygous individuals using a primer from exon 4 and a primer corresponding to *Mu1.7* (primers 1 and 2 as shown in Figure 1B). The *mop1–1* homozygotes give rise to a 470-bp amplicon whereas the WT individuals do not, suggesting that in *mop1–1* homozygotes, the *ZmRDR2* mRNA is transcribed but the transcript includes the *Mu1.7* insertion and is thus nonfunctional. *aat* was used a control.

We have sequenced a portion of a second ethylmethane sulphonate (EMS) allele of *mop1, mop1–2* [28,29]*.* Our sequencing revealed that this allele has a substitution of a G to an A, resulting in a stop codon at amino acid 494 of the deduced protein sequence for the maize RDR2 gene (Figure S1). This polymorphism is unique to *mop1–2;* neither the *mop1–1* allele nor the wild-type *Mop1* allele in our minimal line has this stop codon (unpublished data). Our sequencing data is identical to that obtained by the Chandler laboratory for the *mop1–2* allele. We concluded that the *mop1–2* EMS allele, like *mop1–1,* has a lesion in *ZmRDR2* that would be unlikely to make a functional product. The finding that two independently derived alleles of *mop1* contain unique lesions in *ZmRDR2* demonstrates that *mop1* is indeed an *RDR* homolog.

Many organisms carry several different classes of RDR genes. In *Arabidopsis,* these genes have duplicated and diversified in function, with RDR2 being most closely associated with transposon silencing [19]. To confirm that *Mop1* encodes an ortholog of AtRDR2, and not an ortholog of a different RDR gene, we obtained partial sequences of orthologs of AtRDR2, AtRDR1, and AtRDR6 from rice and maize (Figure S2). These sequences correspond to those flanking the *Mu* insertion depicted in Figure 1A and shown in Figure S1. Phylogenetic analysis of the deduced amino acid sequence of all of these sequences reveals that *ZmRDR2* is more closely related to rice and *Arabidopsis* RDR2 than it is to maize, rice, or *Arabidopsis* RDR1 or RDR6 (Figure S3). Thus we conclude that *ZmRDR2/Mop1* is a true ortholog of *Arabidopsis* RDR2.

#### The *mop1* homozygous mutant produces aberrant mRNA transcripts.

Reverse transcriptase (RT)-PCR using primers that span the *Mu* insertion in *ZmRDR2* failed to amplify in the *mop1–1* homozygous mutants (Figure 2A). However, expression in the *mop1–1* homozygotes was seen when RT-PCR was performed using PCR primers corresponding to exon 3 and the *Mu1.7* TIR, indicating that the produced transcript is aberrant because it contains an unspliced insertion (Figure 2B). The insertion is located in a conserved portion of the gene approximately 220-bp downstream of the RdRP domain (pfam05183) [34] (Figure S2). BLAST searches reveal that sequences flanking the insertion are conserved among RDRs in most species (Figure S2), suggesting that the *mop1–1* mutant allele is unlikely to produce a functional protein.

### *MuDR* Small RNAs Are Not Seen in *mop1–1* Homozygous Mutants

**Figure 3:**
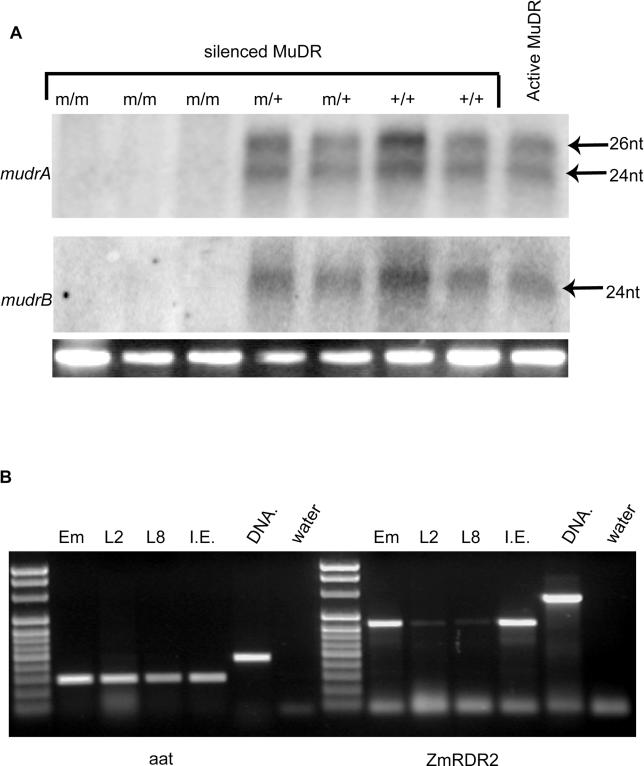
*Mop1* Is Implicated in Small RNA Processing in the Immature Ear (A) Small RNA Northern blot of a family segregating for the *mop1–1* allele. All individuals carrying the WT allele of *Mop1–1* (m/+: *mop1/+* and +/+: WT), including active *MuDR,* contain small 26- and 24-nt RNAs for corresponding to *mudrA* and small 24-nt RNAs corresponding to *mudrB*. None of the individuals that are homozygous for the *mop1–1* mutant (m/m) carry small RNAs corresponding to either *mudrA* or *mudrB,* suggesting that the *Mop1*/*ZmRDR2* gene is involved in the processing of *MuDR* small RNAs. Shown below is a loading control. The same quantity from each RNA sample used for the blot was run in an ethidium-stained gel and photographed. (B) RT-PCR of embryo, leaf, and immature ear cDNA of individuals wild type for *Mop1–1* using primers corresponding to *ZmRDR2*. The right-hand figure depicts much higher levels of *ZmRDR2* transcript in embryo and immature ear tissue than in the leaves (L2, leaf 2; L8, leaf 8). The left-hand picture is the cDNA control using primers specific to aat.

RDR2 has a role in *Arabidopsis* in the maintenance of silencing; when mutated, small RNA transcripts for one SINE retrotransposon, Arabidopsis thaliana short interspersed element 1 *(AtSN1)*, as well as small RNAs for some other transcripts, are lost [19]. We looked at small RNAs corresponding to *mudrA* and *mudrB* in immature ears of *mop1* homozygous mutants and closely related wild-type plants. We focused on immature ears because the expression of *mudrA* and *mudrB* is normally highest in this tissue. We found small RNAs homologous to both *mudrA* (24 and 26 nucleotides [nt]) and *mudrB* (24 nt) in all *mop1* heterozygous and homozygous wild-type individuals, including those that carried an active *MuDR* element (Figure 3A) as well as those that lacked *MuDR* (unpublished data). In contrast, *mop1* homozygous mutant plants lacked small RNAs corresponding to either *mudrA* or *mudrB* (Figure 3A). All maize lines contain at least some sequences that are homologous to *MuDR,* known as *hMuDRs*. These sequences do not appear to contribute to *Mutator* activity, but they do produce some largely nuclear localized transcript [31]. Given that we see small *MuDR*-hybridizing RNAs in plants lacking a functional *MuDR* element, it is likely that these small RNAs are the result of *hMuDR* transcript processing, although silenced *MuDR* elements could also be the source of these small RNAs in plants that carry those elements as well.

Small 24- to 26-nt RNAs that we previously reported [25] as being associated with *Muk*-induced silencing are specific to the first few emerging leaves of plants that carry both *MuDR* and *Muk*. In wild-type plants not undergoing *MuDR* silencing, this young leaf tissue, unlike immature ears, lacks detectable quantities of *MuDR*-homologous 24- to 26-nt small RNAs; in young leaves, these small RNAs are only detected in plants carrying *Muk* or *Muk* with *MuDR* [25]. *ZmRDR2* transcript is present in much lower quantities in leaf tissue compared to either immature ears or embryos (Figure 3B), where we also see small *hMuDR* RNAs (unpublished data), suggesting that the processing of *hMuDR* transcripts may be a function of the availability of *ZmRDR2* gene product in any given tissue. Together with the observation that *MuDR/hMuDR* small RNAs are missing in *mop1* mutants, these data suggest that the production of *MuDR/hMuDR* small RNAs is dependent on the synthesis of dsRNA by the *ZmRDR2* RDR.

### The *mop1* Mutation Does Not Prevent Silencing of *MuDR* by *Muk*

**Figure 4:**
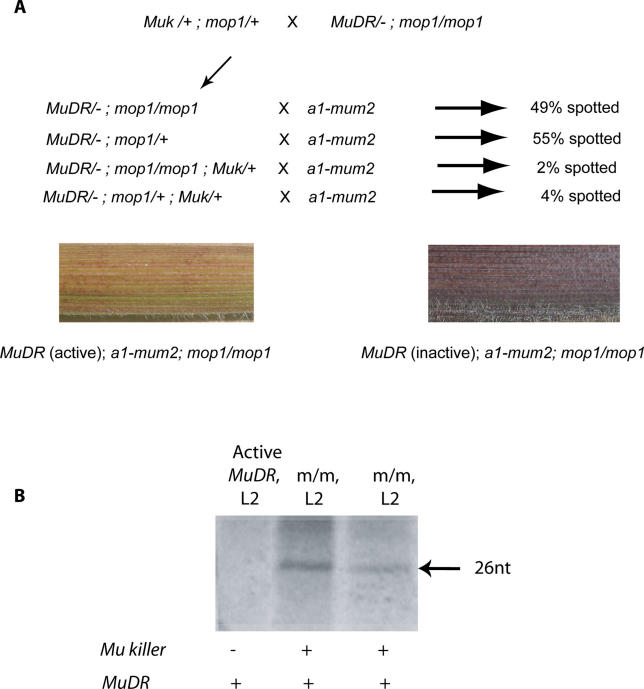
Prevention of *Mutator* Silencing by *Muk* (A) Shown at the top are the progeny of a cross *Muk; mop1/+* × *MuDR/−; mop1/mop1,* which are subsequently crossed to the wild-type *a1-mum2* tester that lacks both *MuDR* and *Muk*. Those individuals that contain *MuDR* but not *Muk* give rise to a significant percentage of spotted kernels when outcrossed; conversely, individuals that carry *Muk* and *MuDR,* regardless of whether they are *mop1* homozygous, give rise to few spotted kernels when outcrossed, suggesting that the *mop1* homozygous mutant does not prevent *Muk* silencing initiation. The images at the bottom show plant color suppression on the left due to active *MuDR* causing suppression of *a1-mum2* expression; on the right is the expected dark plant color indicative of *MuDR* inactivation and lack of *Mutator* suppression at the *a1-mum2* color allele. (B) A small RNA Northern blot of RNA collected from leaf 2 (L2) of individuals heterozygous for active *MuDR*. The individual on the left does not contain *Muk* and is wild-type for *Mop1*. The other two individuals carry *Muk* and are *mop1–1* homozygous mutants (m/m).

The *mop1* homozygous mutant prevents *B′/B-I* paramutation in maize [29]. Because the *mop1* mutant reverses both *Mutator* element methylation and *mudrA* silencing, we wanted to know if it could also prevent the initiation of *MuDR* silencing by *Muk*. To test this, we used genetic analysis to combine *MuDR* with *Muk* in the presence or absence of the *mop1* mutation (Figure 4A). Plants that were homozygous for *mop1* and that carried a single *MuDR* element were crossed reciprocally to and by plants that were heterozygous for both *mop1* and *Muk*. The resulting plants were genotyped for *mop1, Muk,* and *MuDR* (see Materials and Methods) and then test crossed to wild-type testers that lacked *MuDR* and *Muk*. *Mutator* activity was monitored in the kernels of the next generation by the presence of somatic excisions of the nonautonomous *Mu1* element from *a1-mum2,* which are visualized as spots of color on a pale background [32].

If *Mop1* were required for the initiation of a heritable silenced state at *MuDR,* we would expect that *MuDR* would remain active in the progeny of *mop1* mutant plants, which would result in the transmission of roughly 25% heavily spotted kernels (progeny that inherited *MuDR* but not *Muk*). In the presence of the wild-type *Mop1* allele, very few of the progeny kernels of plants that carried *Muk* would be expected to be spotted [8]. We could also examine the plants themselves for evidence of transposase activity by examining *a1-mum2* suppressibility. In the presence of the transposase, *a1-mum2* expression is largely suppressed, resulting in a pale red plant with spots of color due to excision of *Mu1* from *a1-mum2*. In the absence of the transposase, *a1-mum2* expresses and plants are darker red [32]. Plants with silenced *MuDR* elements are consistently darker than plants that carry active *MuDR* due to a lack of suppression of the *a1-mum2* allele (Figure 4A). Thus, it was possible to examine these F1 plants for evidence of *MuDR* activity before analyzing their progeny.

We did not see transmission of active *MuDR* elements from any of the plants that carried *Muk* in this experiment (Table 1). Nine plants that were *mop1* mutants and that carried both *MuDR* and *Muk* gave rise to a total of 2% (8/436) spotted kernels, a percentage consistent with *Muk* successfully silencing *MuDR* [8]. Five sibling plants that were heterozygous for *mop1* and carried *Muk* and *MuDR* gave rise to a total of 4% (37/836) spotted kernels. In contrast, plants that carried *MuDR* but that lacked *Muk* gave rise to 53% (932/1770) spotted kernels, consistent with the segregation of a single *MuDR* element in these families. None of the *mop1* mutant plants that that carried *Muk* and *MuDR* showed evidence of *a1-mum2* suppression, consistent with a lack of *MuDR* activity in these plants.

**Table 1:**
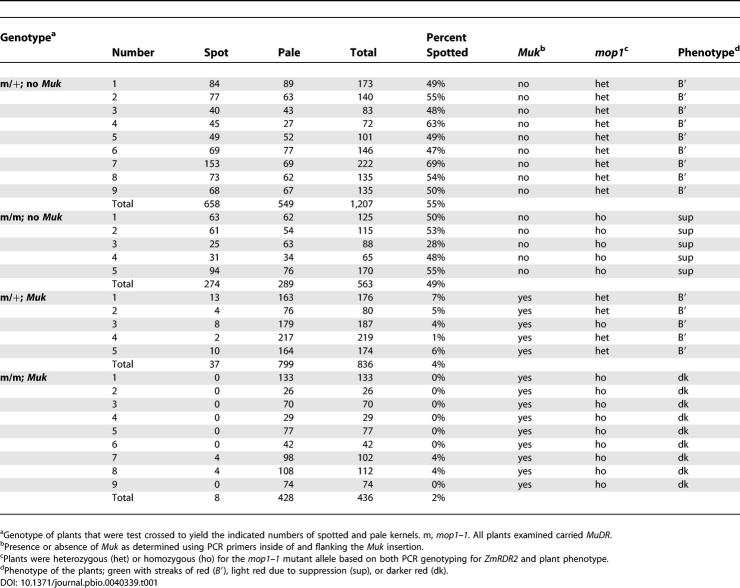
The *mop1–1* Mutant Does Not Prevent *Mutator* Silencing by *Muk*

Clearly, *mop1* did not prevent either the initiation of silencing of *MuDR* in these families, as evidenced by the lack of *a1-mum2* suppression in the F1 plants, or in the establishment of a heritable silenced state, as evidenced by the lack of spotted progeny kernels. This was true regardless of the direction of the original *Muk* cross (Table S1). Because of this, we predicted that the leaf 2–specific small RNA associated with the initiation of silencing of *MuDR* by *Muk* would be still be present in *mop1*/*mop1;Muk;MuDR* leaves, and this is what we observed (Figure 4B). Based on these data, we conclude that *ZmRDR2* is not required for the production of these small RNAs or for the initiation of heritable *Muk*-induced silencing of *MuDR*.

### NAP1 Homolog Knockdown Mutants Prevent Heritable *Muk* Silencing

We obtained a number of publicly available transgenic maize lines from the Chromatin Group at the University of Arizona (ChromDB). These maize lines contain constructs that express inverted repeat portions of genes involved in chromatin remodeling; aside from the inverted repeat sequence that is specific for a particular target gene, these constructs are otherwise identical. Expression of these constructs results in a reduction in the amount of endogenous mRNA from the target gene present in the transgenic plants [35].

**Figure 5:**
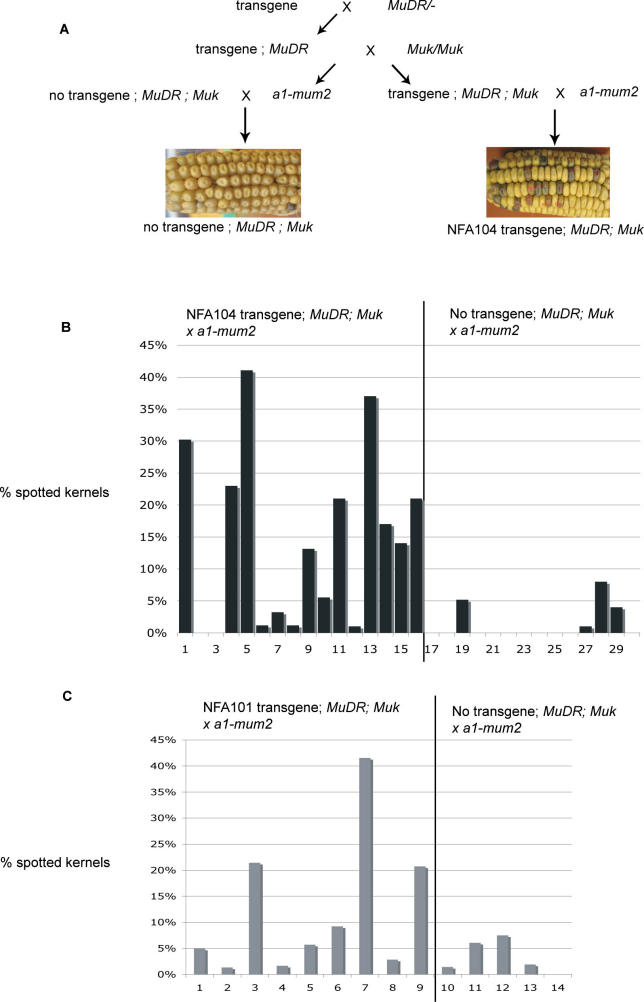
The NAP1 RNAi Mutants Prevent *Mutator* Silencing by *Muk* (A) Diagram of the crosses performed to generate the prophylactic experiment to examine the effects of various RNAi knockdown mutants on the process of *Mutator* silencing by *Muk*. The images are examples of ears derived from plants either lacking (left) or carrying the NFA104 transgene (right). (B) Percent spotted progeny kernels from individuals either carrying the NFA104 transgene (left) or not (right). All individuals carry both *MuDR* and *Muk*. Individuals that carry the NFA104 transgene on average have a higher percentage of spotted kernels compared to individuals that do not, suggesting that the NFA104 transgene can prevent *MuDR* silencing by *Muk*. (C) Percent spotted progeny kernels from individuals either carrying (left) or lacking (right) the NFA101 transgene. All individuals carry both *MuDR* and *Muk*. Individuals that carry the transgene on average have a higher percentage of kernel spotting versus individuals that do not carry the transgene.

We wanted to know if these mutations would affect the process of *Muk*-induced silencing of *MuDR*. To test this, we crossed a selection of plants carrying transgenes targeting one of four different genes to active heterozygous *MuDR* lines: SRT101 (a SIR2-like histone deacetylase), CHR101 (a SWI2/SNF2 chromatin remodeling complex unit), and nucleosome/chromatin assembly factor group A (NFA101 and NFA104, both of which are orthologs of NAP1 nucleosome assembly proteins). We then crossed progeny plants that carried both the transgene and *MuDR* to lines homozygous for *Muk*. Transgenic and nontransgenic individuals that were heterozygous for both *Muk* and *MuDR* were then crossed to wild-type tester lines carrying the *a1-mum2* reporter gene to test for transposon activity (Figure 5A).

**Figure 6:**
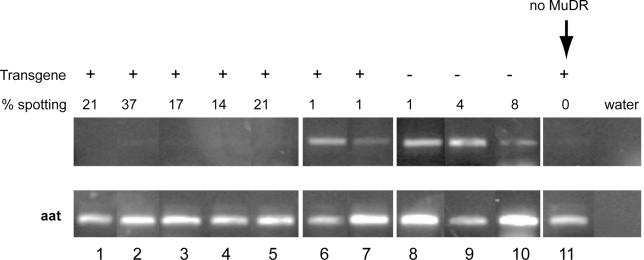
Knockdown of the Endogenous NFA104 Transcript by the NFA104 Transgene Is Correlated with Kernel Spotting in *MuDR;Muk* F1 Progeny RT-PCR of the endogenous NFA104 transcript in individual progeny from the cross NFA104; *MuDR* × *Muk*. NFA104 transgenic individuals (T) (lanes 1–5) in which the endogenous NFA104 transcript has been lost give rise to a significant percentage of spotted kernels (% spotting) when outcrossed to *a1-mum2*. Transgenic individuals where the endogenous NFA104 transcript is present (lanes 6 and 7) give rise to few spotted kernels when crossed to *a1-mum2*. Individuals not carrying the transgene (lanes 8–10) express the endogenous NFA104 transcript and give rise to few spotted kernels when crossed to *a1-mum2*. All individuals carry both *MuDR* (except for lane 11) and *Muk*.

One transgene targeting NFA104 had a pronounced effect on the heritability of *Muk*-induced silencing of *MuDR*. Of the 30 parent individuals of the F1 cross that had both *Muk* and *MuDR*, 16 carried the NFA104 transgene (Figure 5B). When crossed to *a1-mum2* testers, nine out of the 16 transgenic individuals gave rise to a large number of heavily spotted kernels (between 14% and 41%). In contrast, the 14 individuals that were not transgenic gave rise to a much lower proportion of spotted kernels (0% to 8%), most of which were very weakly spotted (Table S2). Kernel spotting is directly correlated with *MuDR* activity; therefore this finding demonstrates that silencing of the NFA104 endogenous gene prevents full heritable *MuDR* silencing by *Muk*. Transgenic individuals that did not prevent *Muk* silencing also failed to exhibit a loss of endogenous NFA104 gene transcript, as demonstrated by RT-PCR (Figure 6), and all of the plants that had a confirmed knockdown of the target gene gave rise to a high frequency (17% to 37%) of spotted progeny kernels. Thus, the variability we see in the heritability of activity in the transgenic class of crosses is likely due to variations in the degree to which the target gene is down-regulated by the transgene.

In contrast to this finding, the two other transgenes, SRT101 and CHR101, which carry identical constructs to NFA104 except for the target sequence, had no effect on *Muk* silencing (Table S3). Because we have not established that SRT101 or CHR101 exhibited successful knockdown of their target genes, we cannot conclude that these targets are not required for silencing. However, these results do demonstrate that the prevention of *Muk* silencing by NFA104 is not due to the generic presence of the construct but rather due to the effect of the NFA104 knockdown on the endogenous NFA104 gene.

### NFA104 Does Not Prevent Loss of *mudrA* Activity in F1 Plants

**Figure 7:**
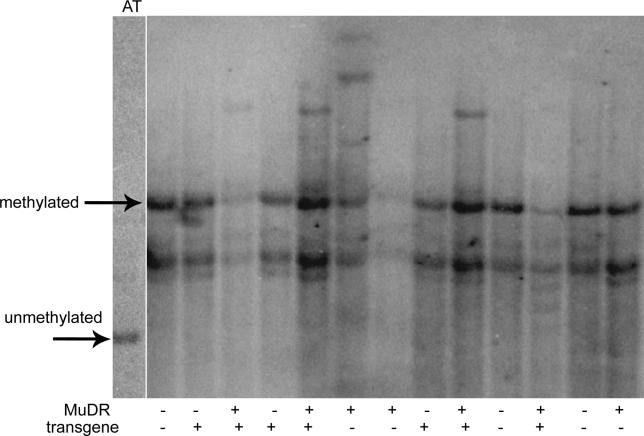
NFA104 Does Not Prevent Methylation of *Mu1* TIRs in a *Muk* Background Shown is a Southern blot of DNA from individuals segregating for the NFA104 transgene (T) and *MuDR* (*), digested with methyl-sensitive *Hin*fI and probed with a *Mu1* fragment. All individuals carry *Muk*. AT, active tester control. Bands at the top of the blot represent *Mu1* insertions that occurred in a previous generation.

To study the possible effects of NFA104 knockdown on the initiation of *MuDR* silencing, we examined 85 individual F1 parents segregating for *MuDR, Muk,* and the NFA104 transgene for evidence of *Mu1* methylation. *Mu1* methylation is a reliable indicator of the absence of *MuDR* transposase *(mudrA);* in active *MuDR* lines, *Mu1* TIRs are not methylated [36]. When *mudrA* is lost via genetic segregation or deletions within *MuDR, Mu1* TIRs are invariably methylated [32,37]. All 54 individuals that carried both *MuDR* and *Muk* exhibited methylation of the *Mu1* TIRs, whether the NFA104 transgene was present (38/54) or absent (16/54) (Figure 7). Together with the test cross data from these plants, this finding suggests that although NFA104 prevents the establishment of a heritably transmitted silenced chromatin state, it does not prevent the initiation of silencing of *MuDR* by *Muk*.

### A Second NFA Transgene Prevents the Full Establishment of *Muk*-Induced Silencing of *MuDR*

Of the five transgenes we examined, only one besides NFA104 prevented *MuDR* silencing by *Muk;* this transgene was another NAP1 homolog, NFA101. Although both NFA101 and NFA104 are homologous to NAP1, they are not the result of a recent duplication; their amino acid sequences are only 27% identical and 48% similar. The results of the F1 cross were similar to those using NFA104: individuals with the transgene gave rise to a much higher number of spotted kernels than those that lacked the transgene (Figure 5C and Table S4). The fact that both NFA/NAP1 transgenes were capable of preventing heritable *MuDR* silencing by *Muk* suggests that this class of genes plays a role in the establishment of *Mutator* silencing in maize.

### Maize NAP1 Homologs Do Not Reactivate Previously Silenced *MuDR* Elements

Because NAP1 mutants reactivate silenced transposons in Caenorhabditis elegans [38], we wanted to see whether knockdown of the NAP1 endogenous genes would reactivate *MuDR* elements silenced by *Muk*. To do this, plants that carried silenced *MuDR* that had lost *Muk* due to genetic segregation were crossed to NFA104 and NFA101 transgenic plants. Plants carrying the transgenes and a silenced *MuDR* element were then test crossed and the progeny kernels examined for somatic activity. Of four individuals carrying both silenced *MuDR* and the NFA104 transgene and six individuals carrying both silenced *MuDR* and the NFA101 transgene, none gave rise to any spotted kernels (0/792 and 0/980, respectively), indicating that the NAP1 genes are not involved in the maintenance of *Mutator* silencing in maize but only in its establishment. Although it is possible that prolonged exposure to the NAP1 transgene would eventually affect *MuDR* silencing (as was seen in *mop1* mutants), none of these mutant plants carried hypomethylated *Mu1* elements (unpublished data), unlike *mop1* mutants, which cause immediate hypomethylation of *Mu1* TIRs whether or not *MuDR* is present.

## Discussion

*Mop1* is the first gene to be cloned in a series of genes implicated in both paramutation [29,33,39] and *Mutator* silencing [27,28]. The discovery that *Mop1* is an RDR2 ortholog demonstrates that both processes are regulated at least in part by RNA processing [33]. However, there are differences between the silencing mechanisms of *Mutator* and paramutation. For instance, whereas the *mop1* mutation does not prevent silencing of *Mutator* transposons, it does prevent paramutagenic silencing of *B-I* by *B′* [29]. This may be explained if the *B′* allele does not produce a dsRNA hairpin product. In that case, dsRNA production in *B-I* silencing initiation would depend on the RDR2 protein. Conversely, *Muk*'s dsRNA hairpin transcript renders the need for RDR2 in *Mutator* silencing initiation unnecessary. *rdr2* mutants in *Arabidopsis* also prevent *FWA* transgene silencing, which, like *B′* paramutation, does not include a dsRNA hairpin [20]. Therefore it appears that the RDR2 protein is necessary for silencing initiation only in circumstances where a dsRNA hairpin is lacking, and RDR activity is therefore required to produce a dsRNA.

In addition to the role of RDR2 in initiation of silencing at loci that do not appear to produce dsRNA on their own, RDR2 is also required for the stable inheritance of the silenced state at several loci in *Arabidopsis* [19]. Similarly, *Mop1 (ZmRDR2)* is required to maintain the silenced state at several paramutant loci in maize as well as transcriptionally silenced transgenes [30], and it is also required to maintain the default methylation at nonautonomous *Mu* element TIRs in the absence of the transposase [28]. However, although *mop1* reverses methylation at the TIR adjacent to the *mudrA* gene, it takes at least five generations in a *mop1* homozygous mutant background to reactivate *mudrA* expression to near-normal levels [27]; a cumulative effect can also be observed with transcriptionally silenced transgenes in the presence of the *mop1* mutation [30]. Thus, the loss of *MuDR*-homologous siRNAs in a *mop1* background causes only a slight destabilization of the silenced chromatin conformation at *mudrA*, but this conformation becomes less stable through multiple cycles of meiosis in the continued absence of siRNAs. It is worth noting that, with the exception of AtSN1, most silenced transposons are not immediately reactivated in an *rdr2* mutant background in *Arabidopsis* [40]. It would be interesting to see if, like *MuDR* in a *mop1* background, these *Arabidopsis* transposons would become reactivated after several generations in the absence of RDR2 product.

The presence of small RNAs that hybridize to both *mudrA* and *mudrB* probes in all wild-type individuals suggests that *hMuDR* and/or *MuDR* transcripts are being processed regardless of the *MuDR* activity. The absence of these small RNAs in *mop1* mutant individuals demonstrates that this processing is dependent on *ZmRDR2*. We suggest that these small RNAs are the result of the maintenance of *hMuDR* silencing, which continues to occur even in the presence of *mudrA* transposase. Although these small RNAs may be important in the maintenance of *hMuDR* silencing, they appear to have no effect on *MuDR* activity in the absence of *Muk*. This may be because these small RNAs only play a role in reinforcing a preexisting chromatin state that does not exist at an active *MuDR* element. Once *Muk* has silenced *MuDR*, small RNAs from either *hMuDRs* or from the silenced element itself may be required for the stability of silencing.

The presence of small RNAs homologous to *mudrB* is somewhat surprising, given that only *mudrA* from a silenced *MuDR* element becomes active in a *mop1* mutant background and that hypomethylation of *MuDR* in a *mop1* mutant background is restricted to the TIR adjacent to *mudrA* [27]. It is likely that another silencing pathway exists for *mudrB* that renders the MOP1 protein redundant, although it is clearly necessary for the production of *hMuDR mudrB* small RNAs.

Our observation that *hMuDR* small RNAs are restricted to tissues in which *ZmRDR2* expresses at a high level (Figure 3B) raises some interesting questions regarding the regulation of transposable elements. Analysis in *Arabidopsis* has revealed that RDR2 expression is much higher in floral tissue than it is in leaves in *Arabidopsis* [19]. Similarly, we see high levels of both *ZmRDR2* expression and *hMuDR* small RNAs in the embryo and immature ears. Because DNA damage caused by transposon activity would be particularly severe and the changes would be heritable in these tissues, it is reasonable that the machinery for silencing transposons would be up-regulated in these tissues as well. It is possible that the high level of expression of *ZmRDR2* in embryos and immature ears versus that of leaves represents a mechanism to reinforce silencing information specifically in undifferentiated cells, and thus may play a role in the regulation of epigenetic information before tissue differentiation. It will be interesting to see a more detailed analysis of the tissue specificity of RDR2 and what role it plays, if any, in cell differentiation.

Although the *mop1–1* mutant does not prevent the establishment of the heritable silencing of *MuDR* by *Muk,* the NAP1 knockdown mutant clearly does. The NAP1 gene has been implicated in chaperoning H2A-H2B histone dimers in yeast [41] and histone H1 in *Drosophila* [42]. Exchange of canonical H2A with variants such as H2AX during nucleosome assembly has been linked with heterochromatin formation [43], and modified forms of histone H1 are associated with the silenced chromatin state of mammals [44]. Further, in *Drosophila*, NAP1 interacts genetically with ACF1 (ATP-utilizing chromatin assembly factor 1) [45] to form repressive chromatin. Given these observations, it is reasonable that NAP1 should also be involved in the establishment of heritable transposon silencing in maize.

We have now identified factors that are necessary for the initiation, establishment, and maintenance of *MuDR* silencing. *Muk* initiates silencing of *Mutator* by targeting the 5′ region of *mudrA* through an RNAi pathway triggered by the hairpin structure of the *Muk* transcript, whereupon *mudrA* mRNA is lost and TIRs become methylated. This process involves the transient production of 24- to 26-nt small RNAs [25], even in the absence of *ZmRDR2* gene activity (Figure 4B). Silencing of *mudrA* in the first generation is also associated with the loss of polyadenylated *mudrB* [8]. It is not known whether alterations in the *mudrB* transcript require an RNA intermediate, but the fact that this only happens in *cis* argues for a chromatin-based spreading process akin to SWI6-mediated spreading at the yeast mating type locus [46]. The establishment of a heritable silenced state does not require *ZmRDR2*, but it does require two maize NAP1 homologs: NFA101 and NFA104. This suggests that these NAP1 homologs are required to establish a form of heritable heterochromatin, perhaps by recruiting specific histone variants. Once established, that silenced state does not appear to require NFA101 or NFA104. Our observation that heritable silencing of *MuDR* by *Muk* does not require *ZmRDR2* activity demonstrates that chromatin modification at *MuDR* that leads to heritable silencing is probably independent of the DCL3/RDR2/AGO4 pathway. Finally, maintenance of *MuDR* silencing is assisted by the *Mop1*/*ZmRDR2* component via an siRNA pathway that is required for RNA-directed DNA methylation. The fact that the *mop1* mutant only gradually reactivates silenced *MuDR* elements, despite the observation that *mop1* mutant invariably and rapidly loses *Mu1* TIR methylation and *MuDR* small RNAs, suggest that *ZmRDR2* activity can act to reinforce a preexisting chromatin state, which can be destabilized if the small RNAs are lacking for several generations.

This model of the silencing mechanism of *Mutator* transposons, although certainly incomplete, provides a clear framework for understanding the progression of naturally occurring transposon silencing. It encompasses factors that specifically influence the initiation *(Muk)*, the establishment (NAP1/NFA), and the maintenance *(Mop1/ZmRDR2)* of *MuDR* silencing. This analysis complements more global analysis of transposon silencing and reactivation, because it suggests that observed variation in epigenetic regulation of transposons [40] can be a function of silencing history as well as position and type of element. As other mutants implicated in both *Mutator* silencing and paramutation are cloned, it will be interesting to see the similarities and differences between these systems and what they can tell us about the process of gene silencing in maize and other organisms.

## Materials and Methods

### Plant materials.

The generation of the *mop1–1* mutant lines and *Muk* lines were previously described in [27] and [8], respectively. Both lines are in the Minimal Mutator background, which consists of one *MuDR* element at position 1 (p1) on chromosome 2L, and one *Mu1* element at the A1 color gene (the *a1-mum2* allele) [32]. The *mop1–2* line was provided by the Chandler laboratory and is derived from an EMS-treated W22 color-converted line [29]. The generation of transgenic lines is described in [35]. Upon reception of the transgenic lines, we crossed these individuals to Minimal Mutator line plants heterozygous for active *MuDR*. The progeny of this cross were then crossed to plants homozygous for *Muk*, and the resultant progeny crossed to the *a1-mum2* Minimal Mutator tester line. The *a1-mum2* tester lacks *MuDR* or *Muk* and is homozygous wild-type for *mop1*.

### Southern blotting.

DNA extraction and Southern blotting were performed on mature maize leaf tissue as described in [27]. Briefly, we probed blots of DNA from families segregating for *mop1/+* and wild-type individuals that had been digested with either *Hin*fI (Figure 7) or *Nco*I (Figure 1) with a *Mu1* probe that hybridizes to both *Mu1* and *Mu1.7* nonautonomous elements.

### PCR and genotyping.

Primers used to identify the *Mu* insertion into *ZmRDR2* were designed based on the *Mu1.7* nonautonomous element sequence and the *ZmRDR2*/RDR101 canonical sequence of exon 4 as follows: RDR2 exon 4F (primer 1), 5′TCTCCACCGCCCACTTGAT3′; *Mu1.7* (primer 2), 5′CCCAAGAGCTGTCTCGTATCCGT3′; PCR conditions were 94 °C, 35 s; annealing temperature 55.9 °C, 45 s; elongation temperature 72 °C, 45 s, for 30 cycles, giving rise to a 470-bp amplicon.

For *Muk* genotyping, primers used to genotype for *Muk* spanned the 5′ region of *Muk* that also corresponds to a portion of *MuDR* TIR as well as the flanking sequence corresponding to the *ACM1* gene within which *Muk* resides. The primers were as follows: for *TIRAR,* 5′AGGAGAGACGGTGACAAGAGGAGTA3′; for *12–4R3,* 5′CGGTATGGCGGCAGTGACA3′, with the cycle 94 °C, 38 s; annealing temperature 59.5 °C, 45 s; elongation temperature 72 °C, 1 min, 34 cycles. MuDR genotyping was as per [27].

### *mop1* genotyping.

All plants described as homozygous for *mop1* showed high levels of expression of B′ and hypomethylation of *Mu-*element TIRs, two characteristic features of this mutation. In addition, all plants described as *mop1* homozygous or heterozygous were genotyped using the SSR umc1541, which is tightly linked (< 1 cM) to the *mop1* locus. For primer sequences and amplification conditions for umc1541, refer to http://www.maizegdb.org/. Plants examined for the prophylactic experiment described in Figure 4 and Table 1 were also genotyped using primers 3 and 4 illustrated in Figure 1. These primers, which flank the *Mu* insertion, failed to amplify a product only in those individuals that were determined to be *mop1* homozygous based on *B*′ expression levels (19 plants). In contrast, all heterozygotes (20 plants) gave rise to the expected 854-bp amplicon.

### Amplification and sequencing of portions of the *mop1–1* and *mop1–2* alleles.

Sequences at the 5′ end of the *Mu* insertion in the *mop1–1* allele were obtained by amplifying using primers 1 and 2 as described above (Figure 1). Sequences at the 3′ end of the insertion were obtained by amplifying with primer 4 (RDR2 exon 4R 5′ATGGCCAGCAGGGTGTCGCAGAT3′) and primer 5 (TIR3′out: 5′GTCGCGTGCGTCTCCAAAACAG3′) (Figure 1A) using the same amplification conditions. These products were amplified and sequenced twice independently, and each product was sequenced on both strands. Sequences for the *mop1–2* allele were obtained by amplification using nested primers located within the second exon of *ZmRDR2*. Primary amplification was with the following: RDRF12 (5′TCTTTTGGCGAGTGTTCC3′) and RDRR12 (5′ATCCTTTATCCCCAATGTT3′), with the cycle 94 °C, 45 s; annealing temperature 54 °C, 45 s; elongation temperature 72 °C, 1 min, 35 cycles. The products were gel isolated and then reamplified using the following second, nested set of primers: RDRF14 (5′TTGCTTCGATGGATGTGTT3′) and RDRR14 (5′TCCAATTTGTAATGTTCAG3′) using the same amplification conditions. Amplification products from plants homozygous for *mop1–1, mop1–2,* and wild-type *Mop1* from the minimal line were obtained. Products from each overlapping sequencing reaction were assembled and compared using SeqMan (DNASTAR, Madison, Wisconsin, United States). The specific lesion present in *mop1–2* was unique to that sequence; each of the other two sequences encoded good open reading frames throughout the length of the sequenced region. All samples were subjected to 35 rounds of amplification, gel isolated, purified using the Qiaquick Gel Extraction kit (Qiagen, Valencia, California, United States), and sequenced using an Applied Biosystems (Foster City, California, United States) sequencer at the University of California.

### RT-PCR.

RNA extraction from maize embryo, mature leaf, and immature ear tissue was performed using TRIzol reagent (Invitrogen, Carlsbad, California, United States) via manufacturer's recommendations, and the reverse transcriptase (RT) procedure was carried out as described previously [27]. For *mop1–1,* primers spanned both intron 3 of the *ZmRDR2* gene and the *Mu1.7* insertion, giving rise to an 854-bp amplicon for *mop1*/+ and wild-type cDNA and a 1335-bp amplicon for wild-type DNA. RDR2 exon 3 (primer 3), 5′ATGCTCCGGGGGCGATTTAGATG3′; RDR2 exon 4R (primer 4), 5′ATGGCCAGCAGGGTGTCGCAGAT3′, with the cycle 94 °C, 35 s; annealing temperature 61.2 °C, 45 s; elongation temperature 72 °C, 45 s, for 30 cycles. These primers were also used for the RT-PCR portrayed in Figure 3B. For *mop1* cDNA transcripts outside of the *Mu1.7* insert, the *Mu1.7* and RDR2 exon 3 primers shown above were used. Spanning intron 3, a 631-bp amplicon was seen in cDNA from *mop1–1/mop1–1* and *mop1–1*/+ individuals, and a 1112-bp product was seen for DNA containing the *mop1–1* allele.

For NFA104 endogenous cDNA sequence, the primers were as follows: 104 forward, 5′CTACCTTCTTCCCTCCGTCTCC3′; 104 reverse, 5′TCGTCGTCGTCGTCATCATC3′, with PCR cycle 94 °C, 35 s, annealing temperature 60 °C, 30 s, elongation temperature 72 °C, 45 s, for 32 cycles, giving rise to an 804-bp amplicon.

As a loading control, cDNA products were also amplified with primers specific for *aat* (alanine aminotransferase). In our hands, this single copy house-keeping gene provides a more reliable control than does ubiquitin. Amplification was done for 29 cycles using the primers *aat*F (5′ATGGGGTATGGCGAGGAT) and *aat*R (5′TTGCACGACGAGCTAAAGACT). Amplification of *aat* cDNA generates a band of 281 bp, whereas amplification of the DNA produces a band of 454 bp.

### Small RNA Northern blot.

Extraction of low– and high–molecular weight RNA from maize immature ear tissue was carried out as described in [47]. Small RNA gel preparation, transfer, and hybridization were performed as previously described [25]. Probes for *mudrA* and *mudrB* small RNAs were created as described in [8] and corresponded to the 5′ sequence of *mudrA* or the 5′ sequence of *mudrB,* respectively, except for Figure 4B, where a probe corresponding to exon 2 of *mudrA* was used. All small RNA bands were sized using the Decade Marker System by Ambion (Austin, Texas, United States) according to manufacturer's instructions.

### Testing for the presence of the transgene.

The constructs used were derived from the pMCG161 plasmid, containing the *BAR* herbicide resistance gene; plants transformed with this construct are resistant to BASTA (glufosinate herbicide) when applied to leaves [35]. Alternately or in addition, plants were screened for the presence of the transgene via PCR using the following primers designed from the *BAR* DNA sequence: BAR forward, 5′CCGTACCGAGCCGCAGGAAC3′; BAR reverse, 5′ATCTCGGTGACGGGCAGGAC3′, for a 436-bp amplicon with the cycle 94 °C, 35 s; annealing temperature 61.5 °C, 45 s; elongation temperature 72 °C, 30 s, for 30 cycles.

## Supporting Information

Figure S1 Sequences Flanking *Mu* Element Insertion into *ZmRDR2* in the *mop1–1* Allele (A) Blue nucleotides represent the 9-bp target site duplication characteristic of a *Mu* insertion. Green nucleotides are TIR sequences. “n”s represent an unspecified number of nucleotides within the insertion. The 3′ end of this sequence, including the last 19 bp of the *Mu* TIR, the 3′ TIR, and the flanking *ZmRDR* sequences are identical to the *mop1–2* allele with the exception of an additional A at position 33 in the published sequence and a G in place of an A at position 218 in the published sequence.
(B) Partial sequence of the *mop1–2* EMS allele. This sequence is identical to the *mop1–1* W22 allele from nucleotides 304 to 1251. The mutation relative to the wild-type B73 sequence is at position 630 (G to A) in our sequence (in red), and at position 933 in the published sequence. The lesion is in exon 2 of the published *ZmRDR2* gene.
(C) A translation of the *mop1–2* EMS allele in the region of interest. Note the stop codon at amino acid 494 replacing a W with a termination codon.
(12 KB PDF)

Figure S2 An Alignment of a Portion of RDRs from Maize, Rice, and *Arabidopsis*
This particular region, which extends from amino acid 865 to 1106 in the published maize RDR sequence, was used because sequences were available for the maize and rice orthologs of AtRDR2, AtRDR1, and AtRDR6. This region includes amino acids that are conserved between all of these sequences as well as an RDR from Branchiostoma floridae, the Florida lancelet, which serves as an outgroup. The site of the *Mu* insertion in *mop1–1* is indicated by a black triangle just after the first block of conserved amino acids.
(49 KB PDF)

Figure S3 A Phylogenetic Tree of the Sequences Presented in Figure 2
Multiple sequence alignments were performed using the CLUSTALW server available at European Bioinformatics Institute (http://www.ebi.ac.uk/clustalw/) with default parameters. A parsimony tree was generated using PAUP 4.0b10 with default settings and 1000 bootstraps. Bootstrap values are as indicated.
(9 KB PDF)

Table S1 The *mop1–1* Mutant Does Not Prevent *Mutator* Silencing by *Muk,* Regardless of the Directionality of the Cross (88 KB DOC)

Table S2 The NFA104 Transgene Prevents *MuDR* Silencing by *Muk*
(96 KB DOC)

Table S3 Two Other Transgenes Tested Did Not Prevent *MuDR* Silencing by *Muk*
(91 KB DOC)

Table S4 The NFA101 Transgene Inhibits *MuDR* Silencing by *Muk*
(71 KB DOC)

### Accession Numbers

The GenBank (http://www.ncbi.nlm.nih.gov/Genbank) accession numbers for sequences discussed in this paper are: *mop1–2* W22 EMS allele, DQ417754; the *mop1–1 Mu* insertion allele, DQ419917; our sequence of *mop1–1* shown in Figure S1, DQ845347; and the published maize B73 wild-type RDR sequence, DQ417753.

## Abbreviations

AGO4: ARGONAUTE 4
AtSN1: *Arabidopsis thaliana* short interspersed element 1
DCL: DICER-LIKE
dsRNA: double-stranded RNA
EMS: ethylmethane sulphonate
*hMuDR*: heterologous *MuDR*
*Mop1*: *Mediator of paramutation1*
*Mu*: *Mutator*
*Muk*: *Mu killer*
NAP1: nucleosome assembly protein 1
NFA: nucleosome/chromatin assembly factor group A
nt: nucleotide
RDR: RNA-dependent RNA polymerase
RNAi: RNA interference
RT: reverse transcriptase
siRNA: short interfering RNA
SSR: simple sequence repeat
TIR: terminal inverted repeat

## References

[pbio-0040339-b001] Ringrose L, Paro R (2001). Remembering silence. Bioessays.

[pbio-0040339-b002] Ringrose L, Paro R (2004). Epigenetic regulation of cellular memory by the Polycomb and Trithorax group proteins. Annu Rev Genet.

[pbio-0040339-b003] Brink RA, Styles ED, Axtell JD (1968). Paramutation: Directed genetic change. Paramutation occurs in somatic cells and heritably alters the functional state of a locus. Science.

[pbio-0040339-b004] Chandler VL, Eggleston WB, Dorweiler JE (2000). Paramutation in maize. Plant Mol Biol.

[pbio-0040339-b005] Kermicle JL, Eggleston WB, Alleman M (1995). Organization of paramutagenicity in *R-stippled* maize. Genetics.

[pbio-0040339-b006] Sidorenko LV, Peterson T (2001). Transgene-induced silencing identifies sequences involved in the establishment of paramutation of the maize *p1* gene. Plant Cell.

[pbio-0040339-b007] Stam M, Belele C, Dorweiler JE, Chandler VL (2002). Differential chromatin structure within a tandem array 100 kb upstream of the maize *b1* locus is associated with paramutation. Genes Dev.

[pbio-0040339-b008] Slotkin RK, Freeling M, Lisch D (2003). Mu killer causes the heritable inactivation of the *Mutator* family of transposable elements in Zea mays. Genetics.

[pbio-0040339-b009] McClintock B (1961). Further studies of the suppressor-mutator system of control of gene action in maize. Carnegie Institution of Washington Yearbook.

[pbio-0040339-b010] Kavi HH, Xie W, Fernandez HR, Birchler JA (2005). Global analysis of siRNA-mediated transcriptional gene silencing. Bioessays.

[pbio-0040339-b011] Yoder JA, Walsh CP, Bestor TH (1997). Cytosine methylation and the ecology of intragenomic parasites. Trends Genet.

[pbio-0040339-b012] Plasterk RH (2002). RNA silencing: The genome's immune system. Science.

[pbio-0040339-b013] Sijen T, Plasterk RH (2003). Transposon silencing in the Caenorhabditis elegans germ line by natural RNAi. Nature.

[pbio-0040339-b014] Aravin AA, Naumova NM, Tulin AV, Vagin VV, Rozovsky YM (2001). Double-stranded RNA-mediated silencing of genomic tandem repeats and transposable elements in the D. melanogaster germline. Curr Biol.

[pbio-0040339-b015] Lippman Z, Gendrel AV, Black M, Vaughn MW, Dedhia N (2004). Role of transposable elements in heterochromatin and epigenetic control. Nature.

[pbio-0040339-b016] Wu-Scharf D, Jeong B, Zhang C, Cerutti H (2000). Transgene and transposon silencing in Chlamydomonas reinhardtii by a DEAH-box RNA helicase. Science.

[pbio-0040339-b017] Almeida R, Allshire RC (2005). RNA silencing and genome regulation. Trends Cell Biol.

[pbio-0040339-b018] Volpe TA, Kidner C, Hall IM, Teng G, Grewal SI (2002). Regulation of heterochromatic silencing and histone H3 lysine-9 methylation by RNAi. Science.

[pbio-0040339-b019] Xie Z, Johansen LK, Gustafson AM, Kasschau KD, Lellis AD (2004). Genetic and functional diversification of small RNA pathways in plants. PLoS Biol.

[pbio-0040339-b020] Chan SW, Zilberman D, Xie Z, Johansen LK, Carrington JC (2004). RNA silencing genes control de novo DNA methylation. Science.

[pbio-0040339-b021] Herr AJ, Jensen MB, Dalmay T, Baulcombe DC (2005). RNA polymerase IV directs silencing of endogenous DNA. Science.

[pbio-0040339-b022] Zilberman D, Cao X, Johansen LK, Xie Z, Carrington JC (2004). Role of *Arabidopsis* ARGONAUTE4 in RNA-directed DNA methylation triggered by inverted repeats. Curr Biol.

[pbio-0040339-b023] Zilberman D, Cao X, Jacobsen SE (2003). ARGONAUTE4 control of locus-specific siRNA accumulation and DNA and histone methylation. Science.

[pbio-0040339-b024] Herr AJ, Baulcombe DC (2004). RNA silencing pathways in plants. Cold Spring Harb Symp Quant Biol.

[pbio-0040339-b025] Slotkin RK, Freeling M, Lisch D (2005). Heritable transposon silencing initiated by a naturally occurring transposon inverted duplication. Nat Genet.

[pbio-0040339-b026] Lisch D, Girard L, Donlin M, Freeling M (1999). Functional analysis of deletion derivatives of the maize transposon *MuDR* delineates roles for the MURA and MURB proteins. Genetics.

[pbio-0040339-b027] Woodhouse MR, Freeling M, Lisch D (2006). The *mop1* (mediator of paramutation1) mutant progressively reactivates one of the two genes encoded by the *MuDR* transposon in maize. Genetics.

[pbio-0040339-b028] Lisch D, Carey CC, Dorweiler JE, Chandler VL (2002). A mutation that prevents paramutation in maize also reverses *Mutator* transposon methylation and silencing. Proc Natl Acad Sci U S A.

[pbio-0040339-b029] Dorweiler JE, Carey CC, Kubo KM, Hollick JB, Kermicle JL (2000). Mediator of *paramutation1* is required for establishment and maintenance of paramutation at multiple maize loci. Plant Cell.

[pbio-0040339-b030] McGinnis KM, Springer C, Lin Y, Carey CC, Chandler VL (2006). Transcriptionally silenced transgenes in maize are activated by three mutations defective in paramutation. Genetics.

[pbio-0040339-b031] Rudenko GN, Walbot V (2001). Expression and post-transcriptional regulation of maize transposable element *MuDR* and its derivatives. Plant Cell.

[pbio-0040339-b032] Chomet P, Lisch D, Hardeman KJ, Chandler VL, Freeling M (1991). Identification of a regulatory transposon that controls the *Mutator* transposable element system in maize. Genetics.

[pbio-0040339-b033] Alleman ML, Lyudmila LV, Vishwas S, McGinnis KM, Dorweiler JE (2006). An RNA-dependent RNA polymerase is required for paramutation in maize. Nature.

[pbio-0040339-b034] Marchler-Bauer A, Bryant SH (2004). CD-Search: Protein domain annotations on the fly. Nucleic Acids Res.

[pbio-0040339-b035] McGinnis K, Chandler V, Cone K, Kaeppler H, Kaeppler S (2005). Transgene-induced RNA interference as a tool for plant functional genomics. Methods Enzymol.

[pbio-0040339-b036] Martienssen R, Baron A (1994). Coordinate suppression of mutations caused by Robertson's mutator transposons in maize. Genetics.

[pbio-0040339-b037] Lisch D, Chomet P, Freeling M (1995). Genetic characterization of the *Mutator* system in maize: Behavior and regulation of *Mu* transposons in a minimal line. Genetics.

[pbio-0040339-b038] Vastenhouw NL, Fischer SE, Robert VJ, Thijssen KL, Fraser AG (2003). A genome-wide screen identifies 27 genes involved in transposon silencing in C. elegans. Curr Biol.

[pbio-0040339-b039] Hollick JB, Kermicle JL, Parkinson SE (2005). Rmr6 maintains meiotic inheritance of paramutant states in Zea mays. Genetics.

[pbio-0040339-b040] Lippman Z, May B, Yordan C, Singer T, Martienssen R (2003). Distinct mechanisms determine transposon inheritance and methylation via small interfering RNA and histone modification. PLoS Biol.

[pbio-0040339-b041] Levchenko V, Jackson V (2004). Histone release during transcription: NAP1 forms a complex with H2A and H2B and facilitates a topologically dependent release of H3 and H4 from the nucleosome. Biochemistry.

[pbio-0040339-b042] Kepert JF, Mazurkiewicz J, Heuvelman GL, Toth KF, Rippe K (2005). NAP1 modulates binding of linker histone H1 to chromatin and induces an extended chromatin fiber conformation. J Biol Chem.

[pbio-0040339-b043] Jin J, Cai Y, Li B, Conaway RC, Workman JL (2005). In and out: Histone variant exchange in chromatin. Trends Biochem Sci.

[pbio-0040339-b044] Lomberk G, Bensi D, Fernandez-Zapico ME, Urrutia R (2006). Evidence for the existence of an HP1-mediated subcode within the histone code. Nat Cell Biol.

[pbio-0040339-b045] Fyodorov DV, Blower MD, Karpen GH, Kadonaga JT (2004). *Acf1* confers unique activities to ACF/CHRAC and promotes the formation rather than disruption of chromatin in vivo. Genes Dev.

[pbio-0040339-b046] Nakayama J, Rice JC, Strahl BD, Allis CD, Grewal SI (2001). Role of histone H3 lysine 9 methylation in epigenetic control of heterochromatin assembly. Science.

[pbio-0040339-b047] Melquist S, Bender J (2003). Transcription from an upstream promoter controls methylation signaling from an inverted repeat of endogenous genes in *Arabidopsis*. Genes Dev.

