# Supplementary material for: Initiation, Establishment, and Maintenance of Heritable MuDR Transposon Silencing in Maize Are Mediated by Distinct Factors
Source: PLoS Biol. 2006 Sep 12;4(10):e339. doi: 10.1371/journal.pbio.0040339 (PMC1563492; doi:10.1371/journal.pbio.0040339)
Supplement: Supplementary file 1 [file pbio.0040339.sg001.pdf]

A)
TGGTTCTCCACCGCCCACTTGATCCACGCAGACCGTGATCCGCTGAAAGCTCGCAGCCC
CGAGTGCGTCCAGCTGGCCGCTCTGCACTCCATGGCGGTCGACTTCGCCAAGACGGGAG
CTCCAGCCGAGATGCCCCTGGCGCTGAGGCCTCGGGAGTTTCCGGACTTCATGGAACGG
TGGGAGAGGCCAATGTACGTCTCCAACGGCGTCCTCGGCAAGCTCTACCGCGCTGCCCT
GCGGCACGAGGAGGACGCAGAAGCGCTCCTGCCTGCGGGCCCACCGAGCTGCGTGTACG
ACCCGGACCTCGAGGTCGCCGGTTTCGACGAGTTCCTGGACGCAGCGGAGGAGCGGTAC
GAGGCGTACGCCGAGAGGCTGGGCGCCCTGATGACCTACTACTCGGCGGAGCGCGAGGA
CGAGATTCTGACAGGCAACATCAGGAACAAGCTGGTGTACCTGCGGCGAGATAATTGCC
ATTATGGACGAAGAGGGAAGGGGATTCGACGAAATGGAGGCGTTGGCGTTGGCTTCTCT
GTTTTGGAGACGCACGCGACAGCCAAACTCCAAAACGGATACGAGACAGCTTCTTGGGn
nnnnnnTCCCCTTCCCTCTTCGTCCATAATAGCAATTATCTCACCTGCGGCGGGACAAC
AAGCGCTACTTCGAGATGAAGGACCGGATCATCGCCGCCGTCGACGCGCTGCACGCGGA
GGTGAGGGGGTGGCTCAGGGCGTGCAAGGAGGACGACGCGTCGAGGGTGGCGTCGGCGT
GGTACCACGTGACCTACCACCCGGACCGCCGCGGGGAGAAGCGGTTCTGGAGCTTCCCG
TGGATCATCTGCGACACCCTGCTG

B)
AATGGCTTTTCCGTGCCTTATGAAGTACTTTTTCGTCTGAACTCTCTTGTTCACATGGG
GAAGCTAGTTGCCAGGCATGTTAATGCTGATCTGTTTAAAGTCCTTGAAGATCTGTCAA
TCGATACTTTAAGGAGAATATTTGAGAAAATGAGCAAATTAAAATCTACCTGCTATGAA
CCTTTGCAATTCATCAGACATGAGGCTCATAGCATGAATATGAGAAAGAAAGCCTTGTC
CAACAAGAGGGAGAGTGGAAAATTGATGAGGTGTTACAGAATTCACATCACACCATCAA
AAATATACTGCTTGGGGCCTGAGGAAGAAGTTTCAAATTACGTGGTTAAGTACCATTCT
GAGTATGCTTCTGACTTTGCTCGAGTTACCTTTGTTGATGAAGATTGGAGCAAGCTTTC
TCCCAATGCATTGTCAGCTAGAACTGAACAAGGTTTCTTTTCTAAACCCTTGAAAACTG
GCCTGTATCATCGTATTCTCTCCATTCTAAAAGAAGGATTTTGCATTGGCCCCAAAAAA
TATGAATTTTTGGCCTTTTCAGCAAGCCAGCTTCGTGGTAATTCTGTTTGGATGTTTGC
TTCAAATAGTTCTTTGACTGCTGAGAATATAAGGAGGTGAATGGGCCACTTTGAAGATA
TCCGTTCAGTCTCCAAGTGTGCAGCTAGAATGGGCCAACTGTTTAGCTCCTCCAGGCAA
ACATTTGAAGTCTCATCATATGATGTAGAAGTAATTCCAGATATTGAAGTCACAACTGA
TGGCACTAAATACATATTTTCAGATGGTATCGGGAAGATTTCTACTAGATTTGCCAGAC
AGGTTGCCAAATTAATTGGCTTAGACCCTGCTCATCCTCCTTCTGCTTTTCAAATAAGG
TATGGGGGCTATAAAGGAGTCATCACTATTGACCCTACTTCCTTTTTCAATCTTTCTCT
GCGA

C)
NGFSVPYEVLFRLNSLVHMGKLVARHVNADLFKVLEDLSIDTLRRIFEKMSKLKSTCYE
PLQFIRHEAHSMNMRKKALSNKRESGKLMRCYRIHITPSKIYCLGPEEEVSNYVVKYHS
EYASDFARVTFVDEDWSKLSPNALSARTEQGFFSKPLKTGLYHRILSILKEGFCIGPKK
YEFLAFSASQLRGNSVWMFASNSSLTAENIRR*MGHFEDIRSVSKCAARMGQLFSSSRQ
TFEVSSYDVEVIPDIEVTTDGTKYIFSDGIGKISTRFARQVAKLIGLDPAHPPSAFQIR
YGGYKGVITIDPTSFFNLSLR
