# Supplementary material for: Initiation, Establishment, and Maintenance of Heritable MuDR Transposon Silencing in Maize Are Mediated by Distinct Factors
Source: PLoS Biol. 2006 Sep 12;4(10):e339. doi: 10.1371/journal.pbio.0040339 (PMC1563492; doi:10.1371/journal.pbio.0040339)
Supplement: Supplementary file 2 [file pbio.0040339.sg002.pdf]

```
OsRDR6     1  DIIDFFLKNMISENLGRICNAHVVHADLSEYGAMDEKCIHLAELAATAVDFPKTGKLAIM
ZmRDR6     1  DIVGFFLKNMVNEKLGPISNAHVVHADMSEYGAMDEKCIQLAELAATAVDFPKTGKIVSM
AtRDR6     1  DIIDFFARNLANEQLGTICNAHVVHADRSEYGAMDEECLLLAELAATAVDFPKTGKIVSM
ZmRDR2     1  EIQKHFVSYMINDTLGAISTAHLIHADRDPLKARSPECVQLAALHSMAVDFAKTGAPAEM
OsRDR2     1  EIQKHFVDYMINDSLGAISTAHLIHADRSPLKARSPECLQLATLHSMAVDFAKTGAPAEM
AtRDR2     1  EIHKFFVDYMISDTLGVISTAHLVHADRDPEKARSQKCLELANLHSRAVDFAKTGAPAEM
OsRDR1     1  EVEEYFTNYIVNESLGMIANAHVVFADKEDLKAESSPCIELAKLFSIAVDFPKTGVPALI
ZmRDR1     1  EIQEYFTNYIVNESLGIIANAHVVFTDQERMKAESPPCVQLAKLFSIAVDFPKTGVPALI
AtRDR1     1  EVEEYFANYIVNDSLGIIANAHTAFADKEPLKAFSDPCIELAKKFSTAVDFPKTGVAAVI
Lancet     1  DMIEFFAEYIGSNQLGLIANAHLVHADKEDQGIFSQKCIDLAQLHSDAVDAPKTGQCPEL

OsRDR6    61  PPHLKPKVYPDFMGKEDGQSYKSEKILGRLYRSIQEASNGD-VVS--QEVCTPNDLPYDI
ZmRDR6    61  PASLRPKLYPDFMGKEDAISYRSEKILGRLYRSIQEASSDD-LVP--EETCTSNNLPYDA
AtRDR6    61  PFHLKPKLYPDFMGKEDYQTYKSNKILGRLYRRVKEVYDED-AEASSEESTDPSAIPYDA
ZmRDR2    61  PLALRPREFPDFMERWERPMYVSNGVLGKLYRAALRHEE---DAEALLPAGP-PSCVYDP
OsRDR2    61  PRTLRPREYPDFMERWEKPMYISNGVLGKLYRSAMGHMEKSGDSGALSSSSAQPSPTYDP
AtRDR2    61  PYALKPREFPDFLERFEKPTYISESVFGKLYRAVKSSLA-----QRKPEAESEDTVAYDV
OsRDR1    61  PPELHVKEYPDFMEKLDKVTYESKGVIGKLYREIKKHTP-----HIKHFTREVARRSYDT
ZmRDR1    61  PHELHVKEYPDFMEKLDKVTYESKGVIGKLYREIKKHTP-----HIKHFTREVARRSYDT
AtRDR1    61  PQHLYVKEYPDFMEKPDKPTYESKNVIGKLFREVKERAPP--LISIKSFTLDVASKSYDK
Lancet    61  KGDLRPEVYPDFMMKSDKPQYRSERIIGRLFRKCQELDSAN--QRSGTDDIVYHTSRIDH

OsRDR6   118  DLEVPGASDFIASAWQCKCSYDAQLSALLSQYRVRTEAELVTGHITFLVKNSSKKQG---
ZmRDR6   118  DMEVAGAADFLSSAWQCKCSYETQLNALINQYGVRTEAELVTEHIWSLPKYSSRRQG---
AtRDR6   120  VLEIPGFEDLIPEAWGHKCLYDGQLIGLLGQYKVQKEEEIVTGHIWSMPKYTSKKQG---
ZmRDR2   117  DLEVAGFDEFLDAAEERYEAYAERLGALMTYYSAEREDEILTGNIRNKLVYLRRDNKRYF
OsRDR2   121  DLEVPGSDEFLQAAEEYYELYEEKLTTLMNYYRAELEDEILTGNIRNKMLYLKRDNKRYF
AtRDR2   116  TLEEAGFESFIETAKAHRDMYGEKLTSLMIYYGAANEEEILTGILKTKEMYLARDNRRYG
OsRDR1   116  DMIVDGYEDYITEAMALKDEYDFKLGNLMDHYGIKSEAEIISGCILKMAKNFTKKSD---
ZmRDR1   116  DLIVDGYEDYITEAIEFKEEYDFRLGNLMDHYGIKSEAEIISGCILKMAKNFTKSSD---
AtRDR1   119  DMEVDGFEEYVDEAFYQKANYDFKLGNLMDYYGIKTEAEILSGGIMRMSKSFTKRRD---
Lancet   119  DLVREGFDSYIEDAQTQLNIYNNMMMSIMSLYGITSEGEVVSGCILKVKQRLGLLKNERF

OsRDR6   175  DIKDRLKTAYSALRKEFKSTFESIASDQCEIGDDEKNLLYEMKASAWYQVTYHPKWVEKS
ZmRDR6   175  DIKERLKNAYYALHKEFRSIFESIVTDQTEISDDEKSRFYEMKASAWYQVTYHPEWVQKS
AtRDR6   177  ELKERLKHSYNSLKKEFRKVFEETIPDHENLSEEEKNILYEKKASAWYHVTYHPEWVKKS
ZmRDR2   177  EMKDRIIAAVDALHAEVRGWLR-ACKED----DASR------VASAWYHVTYHP------
OsRDR2   181  EMKDRIVAAVDALHREARGWLLSSRKEE----DASR------MASAWYRVTYHP------
AtRDR2   176  DMKDRITLSVKDLHKEAMGWFEKSCEDE----QQKKK-----LASAWYYVTYNP------
OsRDR1   173  --ADAIRLAVRSLRKEARSRFSEMSLDD----NGHGHDASEAKASAWYHVTYHPEFWGCY
ZmRDR1   173  --ADAIRMAVRSLRKEARSWFNEMSTGE----DGQ--DAMEAKASAWYHVTYHQQYWGSY
AtRDR1   176  --AESIGRAVRALRKETLSLFNASEEEE----NES------AKASAWYHVTYHSSYWGLY
Lancet   179  EVAEYVRARFKTLWRKTRAEFFKQFDVEELS-EERRKEEQLKKASAWYMVTYSDAN----

OsRDR6   235  RGILGPDGEEIPASLSFAWIPVDYLARIK
ZmRDR6   235  REMFKSDCEDMPARLSFAWIAVEHLARIK
AtRDR6   237  LELQDPDESSHAAMLSFAWIAADYLARIK
ZmRDR2   220  -----DRRGEKR-FWSFPWIICDTLLAIK
OsRDR2   225  -----DRRRGKR-FWSFPWIACDNLLAIK
AtRDR2   221  -----NHRDEKLTFLSFPWIVGDVLLDIK
OsRDR1   227  -----NEGYERPHFISFPWCIYEKLLRIK
ZmRDR1   225  -----NEGYDRPHLISFPWCVYDKLVAIK
AtRDR1   224  -----NEGLNRDHFLSFAWCVYDKLVRIK
Lancet   234  -----KPLDPSPKLLSFPWVVYDVLASLK
```
