# Supplementary material for: Initiation, Establishment, and Maintenance of Heritable MuDR Transposon Silencing in Maize Are Mediated by Distinct Factors
Source: PLoS Biol. 2006 Sep 12;4(10):e339. doi: 10.1371/journal.pbio.0040339 (PMC1563492; doi:10.1371/journal.pbio.0040339)
Supplement: Supplementary file 3 [file pbio.0040339.sg003.pdf]

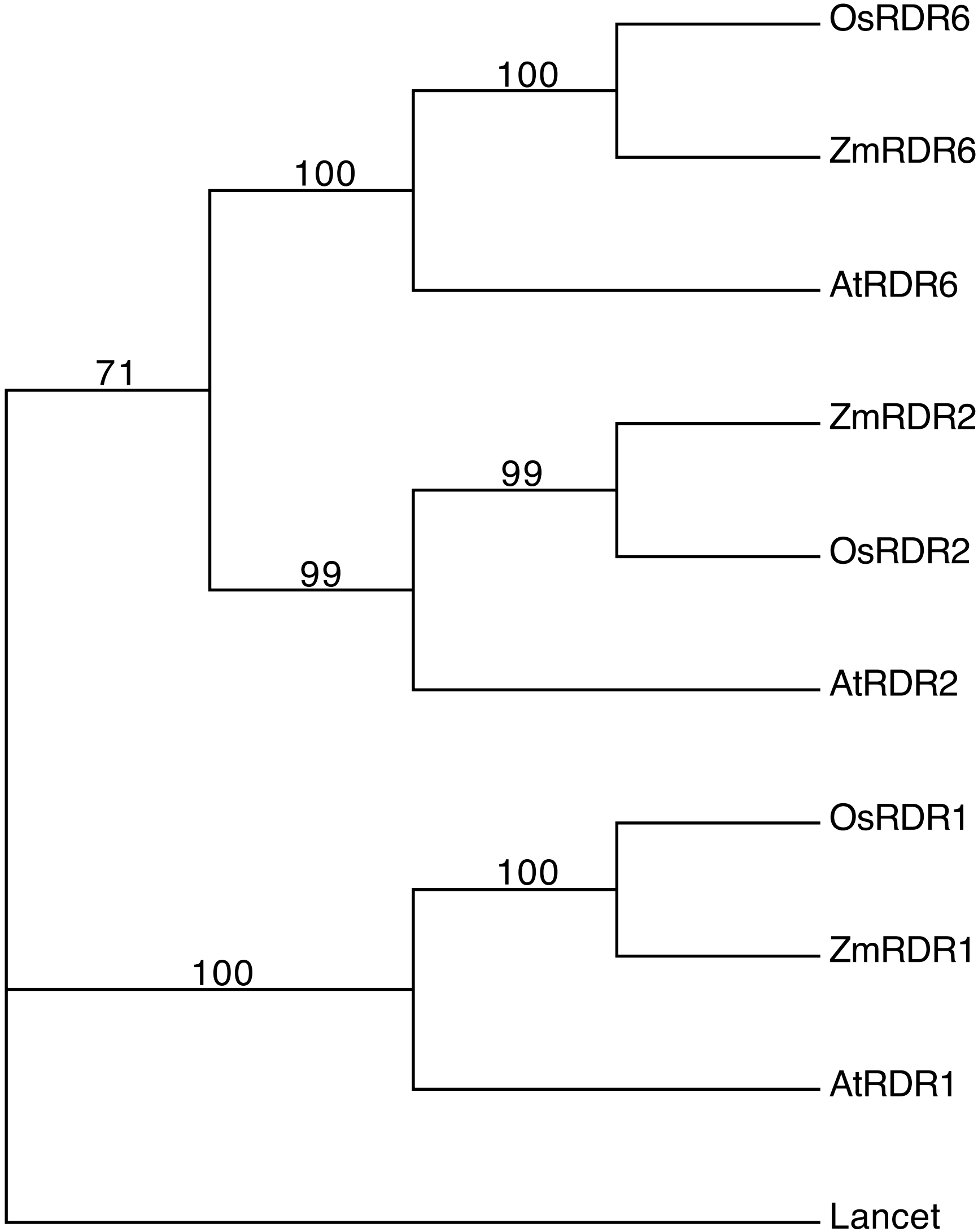
OsRDR6
ZmRDR6
AtRDR6
ZmRDR2
OsRDR2
AtRDR2
OsRDR1
ZmRDR1
AtRDR1
Lancet
100
100
71
99
99
100
100
