# Supplementary material for: Initiation, Establishment, and Maintenance of Heritable MuDR Transposon Silencing in Maize Are Mediated by Distinct Factors
Source: PLoS Biol. 2006 Sep 12;4(10):e339. doi: 10.1371/journal.pbio.0040339 (PMC1563492; doi:10.1371/journal.pbio.0040339)
Supplement: Supplementary file 4 [file pbio.0040339.st001.doc]

**Supplemental Data Table 1**

**The *mop1-1* mutant does not prevent *Mutator* silencing by *Muk,* regardless of the directionality of the cross**

*Progeny of a cross MuDR/-;mop1/mop1 x Muk;mop1/+ crossed to* a1-mum2

|  | Spot | Pale | Total | % spot | *Muk* ^b^ | *mop1* ^c^ | phen. ^d^ |
| --- | --- | --- | --- | --- | --- | --- | --- |
| m/+; no *Muk* |  |  |  |  |  |  |  |
| 1 | 74 | 60 | 134 | 55% | no | het | B' |
| 2 | 99 | 105 | 204 | 49% | no | het | B' |
| total | 173 | 165 | 338 | 51% |  |  |  |
|  |  |  |  |  |  |  |  |
| m/m; no *Muk* |  |  |  |  |  |  |  |
| 1 | 42 | 34 | 76 | 55% | no | ho | sup |
| 2 | 21 | 18 | 39 | 54% | no | ho | sup |
| 3 | 52 | 27 | 79 | 66% | no | ho | sup |
| total | 115 | 289 | 563 | 59% |  |  |  |
|  |  |  |  |  |  |  |  |
| m/+; *Muk* |  |  |  |  |  |  |  |
| 1 | 17 | 177 | 194 | 9% | yes | het | B' |
| 2 | 6 | 47 | 53 | 11% | yes | het | B' |
| 3 | 13 | 135 | 148 | 9% | yes | het | B' |
| 4 | 0 | 137 | 137 | 0% | yes | het | B' |
| total | 36 | 496 | 532 | 7% |  |  |  |
| m/m; no *Muk* |  |  |  |  |  |  |  |
| 1 | 2 | 114 | 133 | 2% | yes | ho | dk |
| 2 | 1 | 238 | 26 | 0% | yes | ho | dk |
| total | 3 | 352 | 355 | 1% |  |  |  |

^a^ genotype of plants that were test crossed to yield the indicated numbers of spotted and pale kernels. m = *mop1-1*; Muk = *Mu killer*. All plants examined carried *MuDR*.

^b^ indicates the presence of absence of *Mu killer* as determined using PCR primers inside of and flanking the *Mu killer* insertion.

^c^ indicates whether the plants were heterozygous or homozygous for the *mop1-1* mutant allele based on both PCR genotyping for *ZmRDR2* and plant phenotype.

^d^ indicates the phenotype of the plants; green with streaks of red (B’), light red due to suppression (sup) or darker red (dk).
