# Supplementary material for: Initiation, Establishment, and Maintenance of Heritable MuDR Transposon Silencing in Maize Are Mediated by Distinct Factors
Source: PLoS Biol. 2006 Sep 12;4(10):e339. doi: 10.1371/journal.pbio.0040339 (PMC1563492; doi:10.1371/journal.pbio.0040339)
Supplement: Supplementary file 5 [file pbio.0040339.st002.doc]

**Supplemental data Table 2**

**The NFA104 transgene prevents *MuDR* silencing by *Muk***

NFA104 transgene; *MuDR; Muk* x *a1-mum2	MuDR; Muk* x *a1-mum2*

| Genotype^a^ | spot | pale | total | %spot | ^b^ |  | Genotype^a^ | spot | pale | total | %spot | ^b^ |
| --- | --- | --- | --- | --- | --- | --- | --- | --- | --- | --- | --- | --- |
| 104 T^c^ |  |  |  |  |  |  | no 104 T^c^ |  |  |  |  |  |
| 1 | 68 | 157 | 225 | 30% | R |  | 1 | 0 | 195 | 195 | 0% | S |
| 2 | 0 | 122 | 122 | 0% | R |  | 2 | 0 | 192 | 192 | 0% | S |
| 3 | 0 | 264 | 264 | 0% | R |  | 3 | 15 | 276 | 291 | 5% | S |
| 4 | 46 | 154 | 200 | 23% | R |  | 4 | 0 | 224 | 224 | 0% | S |
| 5 | 103 | 148 | 251 | 41% | R |  | 5 | 0 | 364 | 364 | 0% | S |
| 6 | 3 | 255 | 258 | 1% | R |  | 6 | 0 | 315 | 315 | 0% | S |
| 7 | 6 | 180 | 186 | 3% | R |  | 7 | 0 | 132 | 132 | 0% | S |
| 8 | 3 | 252 | 255 | 1% | R |  | 8 | 0 | 185 | 185 | 0% | S |
| 9 | 36 | 238 | 274 | 13% | R |  | 9 | 0 | 35 | 35 | 0% | S |
| 10 | 18 | 308 | 326 | 6% | R |  | 10 | 0 | 392 | 392 | 0% | S |
| 11 | 22 | 84 | 106 | 21% | R |  | 11 | 4 | 274 | 278 | 1% | S |
| 12 | 2 | 150 | 152 | 1% | R |  | 13 | 27 | 314 | 341 | 8% | S |
| 13 | 27 | 46 | 73 | 37% | R |  | 14 | 5 | 131 | 136 | 4% | S |
| 14 | 31 | 150 | 181 | 17% | R |  | 15 | 1 | 221 | 222 | 0% | S |
| 15 | 2 | 12 | 14 | 14% | R |  | total | 52 | 3250 | 3302 | 1% |  |
| 16 | 49 | 184 | 233 | 21% | R |  |  |  |  |  |  |  |
| total | 416 | 2704 | 3120 | 14% |  |  |  |  |  |  |  |  |

^a^ genotype of plants that were the result of the indicated cross. All individuals are heterozygous for both *MuDR* and *Mu killer.*

^b^ indicates resistance (R) or sensitivity (S) to Basta herbicide. R individuals are those that carry the transgene.

^c^ 104 T = the NFA104 transgene

Results of a Student’s t-test:

t=3.38

sdev=10.1

df=29

probability assuming null hypothesis: 0.002
