# Supplementary material for: Initiation, Establishment, and Maintenance of Heritable MuDR Transposon Silencing in Maize Are Mediated by Distinct Factors
Source: PLoS Biol. 2006 Sep 12;4(10):e339. doi: 10.1371/journal.pbio.0040339 (PMC1563492; doi:10.1371/journal.pbio.0040339)
Supplement: Supplementary file 6 [file pbio.0040339.st003.doc]

**Supplemental Data Table 3**

**Two other transgenes tested did not prevent *MuDR* silencing by *Mu killer***

| G^a^ | spot | pale | total | %spot | B^b^ |
| --- | --- | --- | --- | --- | --- |
| SRT101 T ^c^ |  |  |  |  |  |
| 1 | 28 | 150 | 178 | 16% | R |
| 2 | 1 | 240 | 241 | 0% | R |
| 3 | 4 | 196 | 200 | 2% | R |
| 4 | 0 | 240 | 240 | 0% | R |
| 5 | 23 | 266 | 289 | 8% | R |
| total | 56 | 1092 | 1148 | 5% | total |
|  |  |  |  |  |  |
| no SRT101 T ^c^ |  |  |  |  |  |
| 1 | 3 | 107 | 110 | 3% | S |
| 2 | 3 | 288 | 291 | 1% | S |
| 3 | 4 | 216 | 220 | 2% | S |
| 4 | 11 | 210 | 221 | 5% | S |
| 5 | 0 | 336 | 336 | 0% | S |
| 6 | 2 | 372 | 374 | 1% | S |
| total | 23 | 1529 | 1552 | 1% | total |
|  |  |  |  |  |  |
| CHR101 T ^c^ |  |  |  |  |  |
| 1 | 8 | 288 | 296 | 3% | R |
| 2 | 1 | 97 | 98 | 1% | R |
| 3 | 2 | 160 | 162 | 1% | R |
| 4 | 0 | 73 | 73 | 0% | R |
| 5 | 0 | 264 | 182 | 0% | R |
| 6 | 1 | 237 | 168 | 1% | R |
| 7 | 1 | 73 | 74 | 1% | R |
| total | 13 | 1192 | 1205 | 1% | total |
|  |  |  |  |  |  |
| no CHR101 T ^c^ |  |  |  |  |  |
| 1 | 4 | 182 | 186 | 2% | S |
| 2 | 3 | 93 | 96 | 3% | S |
| 3 | 2 | 188 | 190 | 1% | S |
| total | 9 | 463 | 472 | 2% | total |

^a^ genotype of plants that were the result of the indicated cross. All individuals are heterozygous for both *MuDR* and *Mu killer.*

^b^ indicates resistance (R) or sensitivity (S) to Basta herbicide. R individuals are those that carry the transgene.

^c^ indicates the transgene
