# Supplementary material for: Initiation, Establishment, and Maintenance of Heritable MuDR Transposon Silencing in Maize Are Mediated by Distinct Factors
Source: PLoS Biol. 2006 Sep 12;4(10):e339. doi: 10.1371/journal.pbio.0040339 (PMC1563492; doi:10.1371/journal.pbio.0040339)
Supplement: Supplementary file 7 [file pbio.0040339.st004.doc]

**Supplemental Data Table 4**

**The NFA101 transgene inhibits *MuDR* silencing by *Muk***

NFA101 transgene; *MuDR; Muk* x *a1-mum2	MuDR; Muk* x *a1-mum2*

| Genotype^a^ | spot | pale | total | %spot | B^b^ |  | Genotype^a^ | spot | pale | total | %spot | B^b^ |
| --- | --- | --- | --- | --- | --- | --- | --- | --- | --- | --- | --- | --- |
| 101 T^c^ |  |  |  |  |  |  | no 101 T^c^ |  |  |  |  |  |
| 1 | 18 | 348 | 366 | 5% | R |  | 1 | 2 | 138 | 140 | 1% | S |
| 2 | 4 | 297 | 301 | 1% | R |  | 2 | 19 | 294 | 313 | 6% | S |
| 3 | 18 | 66 | 84 | 21% | R |  | 3 | 21 | 260 | 281 | 7% | S |
| 4 | 5 | 300 | 305 | 2% | R |  | 4 | 4 | 206 | 210 | 2% | S |
| 5 | 13 | 216 | 229 | 6% | R |  | 5 | 0 | 240 | 240 | 0% | S |
| 6 | 27 | 266 | 293 | 9% | R |  | total | 46 | 1138 | 1184 | 3% |  |
| 7 | 44 | 62 | 106 | 42% | R |  |  |  |  |  |  |  |
| 8 | 3 | 103 | 106 | 3% | R |  |  |  |  |  |  |  |
| 9 | 46 | 176 | 222 | 21% | R |  |  |  |  |  |  |  |
| total | 178 | 1834 | 2012 | 12% |  |  |  |  |  |  |  |  |

^a^ genotype of plants that were the result of the indicated cross. All individuals are heterozygous for both *MuDR* and *Mu killer.*

^b^ indicates resistance (R) or sensitivity (S) to Basta herbicide. R individuals are those that carry the transgene.

^c^ 101 T = the NFA101 transgene.

Results of a Student’s t-test:

t=1.45

sdev=11.2

df=12

Probability assuming null hypothesis: 0.173
